# Regulatory Potential of bHLH-Type Transcription Factors on the Road to Rubber Biosynthesis in *Hevea brasiliensis*

**DOI:** 10.3390/plants9060674

**Published:** 2020-05-26

**Authors:** Tomoko Yamaguchi, Yukio Kurihara, Yuko Makita, Emiko Okubo-Kurihara, Ami Kageyama, Emi Osada, Setsuko Shimada, Hiroko Tsuchida, Hiroaki Shimada, Minami Matsui

**Affiliations:** 1 Synthetic Genomics Research Group, RIKEN Center for Sustainable Resource Science, Yokohama, Kanagawa 230-0045, Japan; 8318567@alumni.tus.ac.jp (T.Y.); yukio.kurihara@riken.jp (Y.K.); yuko.makita@riken.jp (Y.M.); emiko.kurihara@riken.jp (E.O.-K.); ami.kageyama@riken.jp (A.K.); emi.osada@riken.jp (E.O.); sshimada@riken.jp (S.S.); hiroko.tsuchida@riken.jp (H.T.); 2 Department of Biological Science and Technology, Tokyo University of Science, Katsushika, Tokyo 125-8585, Japan; shimadah@rs.noda.tus.ac.jp; 3 Graduate School of Nanobioscience, Department of Life and Environmental System Science, Yokohama City University, Yokohama, Kanagawa 236-0027, Japan

**Keywords:** bHLH, transcription factor, rubber, jasmonate

## Abstract

Natural rubber is the main component of latex obtained from laticifer cells of *Hevea brasiliensis*. For improving rubber yield, it is essential to understand the genetic molecular mechanisms responsible for laticifer differentiation and rubber biosynthesis. Jasmonate enhances both secondary laticifer differentiation and rubber biosynthesis. Here, we carried out time-course RNA-seq analysis in suspension-cultured cells treated with methyljasmonic acid (MeJA) to characterize the gene expression profile. Gene Ontology (GO) analysis showed that the term “cell differentiation” was enriched in upregulated genes at 24 h after treatment, but inversely, the term was enriched in downregulated genes at 5 days, indicating that MeJA could induce cell differentiation at an early stage of the response. Jasmonate signaling is activated by MYC2, a basic helix–loop–helix (bHLH)-type transcription factor (TF). The aim of this work was to find any links between transcriptomic changes after MeJA application and regulation by TFs. Using an in vitro binding assay, we traced candidate genes throughout the whole genome that were targeted by four bHLH TFs: Hb_MYC2-1, Hb_MYC2-2, Hb_bHLH1, and Hb_bHLH2. The latter two are highly expressed in laticifer cells. Their physical binding sites were found in the promoter regions of a variety of other TF genes, which are differentially expressed upon MeJA exposure, and rubber biogenesis-related genes including *SRPP1* and *REF3*. These studies suggest the possibilities that *Hb_MYC2-1* and *Hb_MYC2-2* regulate cell differentiation and that *Hb_bHLH1* and *Hb_bHLH2* promote rubber biosynthesis. We expect that our findings will help to increase natural rubber yield through genetic control in the future.

## 1. Introduction

Natural rubber, which is highly industrially important, is a biomass material made of cis-1,4-polyisoprene and the main component of latex obtained from laticifer cells of the rubber tree, *Hevea brasiliensis* [1]. Natural rubber has a superior mechanical strength, including abrasion resistance, compared to synthetic rubber, and is indispensable for aircraft tires, etc. [2]. With the rapid economic growth of emerging nations, the demand for natural rubber is growing year by year [3]. Therefore, improvement in the natural rubber yield per tree is essential.

Laticifer cells are present around the vascular bundle behind the bark. In these cells, isopentenyl diphosphate (IPP), a precursor of cis-1,4-polyisoprene, is synthesized from sucrose sent from the leaves via the cytosolic mevalonate (MVA) pathway or the plastidic 2-C-methyl-D-erythritol-4-phosphate (MEP) pathway [4]. Of the two, the MVA pathway is the main contributor of IPP for rubber biosynthesis [3,5]. IPP is polymerized into cis-1,4-polyisoprene on the rubber particle membranes by rubber biosynthetic proteins including cis-prenyltransferase (CPT), CPT-like protein (CPTL, also known as a Nogo-B receptor), rubber elongation factor (REF), and small rubber particle protein (SRPP) [3,6,7,8,9,10]. Previous tissue-specific RNA-seq analysis showed that, in concert with their activity in laticifer cells, the expression level of these proteins is the highest in latex (laticifer cells) compared with petiole, bark, and leaf [11]. However, the regulatory mechanism for the laticifer cell-specific expression of rubber biosynthetic genes still remains to be elucidated.

Natural rubber yield is correlated with the number of laticifer cells. It is well-known that application of jasmonate induces laticifer cell differentiation and promotes rubber biosynthesis in *H. brasiliensis* [12]. Previous works performed transcriptome analyses on rubber trees treated with jasmonate or coronatine, a functional mimic of active jasmonate, and revealed several molecular insights, such as the involvement of some TFs in laticifer differentiation and rubber biosynthesis [13,14,15,16,17]. However, they did not identify any apparent determinants for the biological processes because they have not shown which genes are directly regulated by the TFs.

*MYC2*, encoding a bHLH-type TF, is a major regulator of the jasmonate response [18,19]. In *Arabidopsis thaliana*, MYC2 and the relevant MYC3 and MYC4 induce expression of jasmonate-responsive genes by binding to G-box-like motifs in the promoters of direct target genes [19,20,21,22]. It has been shown that, in the rubber tree, the expression level of MYC2 is increased by jasmonate treatment, suggesting that MYC2 is also an important factor in the jasmonate response in *H. brasiliensis* [23]. 

Transcriptional regulation plays a crucial role in controlling gene expression in plants. In particular, typical TFs mediate multiple biological processes by activating or inactivating the transcription of the genes [24]. It is essential to identify TFs that regulate genes required for laticifer differentiation and rubber biosynthesis. For this purpose, the high-throughput in vitro genomic DNA-binding sequencing (gDB-seq) method is useful [25,26]. It can identify genome-wide physical TF binding sites more easily and in a shorter time period than the in vivo assay, chromatin-immunoprecipitation sequencing (ChIP-seq). 

In this study, we aimed to elucidate the transcriptional regulation of genes involved in jasmonate signaling possibly associated with laticifer differentiation and rubber biosynthesis. Using RNA-seq analysis, we characterize the gene expression profile in suspension-cultured cells derived from *H. brasiliensis* upon methyljasmonic acid (MeJA) treatment. Furthermore, using gDB-seq analysis, we identify physical binding sites of four bHLH-type TFs; two of them are homologous with *AtMYC2* and the others show high expression in laticifer cells. By combining the results of the RNA-seq and gDB-seq analyses, we expect to understand more deeply the transcriptional regulation of jasmonate signaling and rubber biosynthesis.

## 2. Materials and Methods 

### 2.1. Suspension-Cultured Cells, Treatment with MeJA or DMSO 

Suspension cells were generated from petioles of *H. brasiliensis* RRIM 600 progeny via callus formation. The cells were diluted 20-fold with modified AA medium [27] at weekly intervals. The modified AA medium contained 0.2 mg/L 2.4-D and 0.2 mg/L kinetin instead of the original concentrations. The cell suspension was agitated on a rotary shaker at 130 rpm at 30 °C in the dark. Two or three microliters of 1mM MeJA dissolved in DMSO or the equivalent amount of DMSO were added to 2 mL or 3 mL of cultured cells immediately after the weekly dilution for 4 h and 24 h samples or 5 d samples, respectively. The treated samples were harvested at 4 h, 24 h, and 5 d after the addition of MeJA or DMSO. The non-treated samples (2 mL) were harvested just after dilution. 

### 2.2. RNA Extraction, RNA-Seq and the Analysis

Total RNA was extracted from a sample using an RNeasy Plant Mini Kit (QIAGEN). RNA-seq libraries were constructed using a TruSeq Stranded mRNA Library Prep Kit (Illumina, San Diego, CA, USA) according to the manufacturer’s instructions and paired-end sequenced (2 × 150 bp) using a HiSeq4000 (Illumina). Quality control (Q > 20) was performed against raw read data and the resulting reads were mapped onto the *H. brasiliensis* RRIM 600 genome [26,28] using STAR version 2.7.0f [29]. Three biological replicates for each condition were performed. FPKM (fragments per kilobase of exon per million reads mapped) values were calculated using Cufflinks version 2.2.1 [30]. Significant differences between MeJA-treated and DMSO-treated samples were defined by Welch’s *t* test (*p* < 0.05), FPKM average > 0.1 and fold difference > 1.5.

Gene Ontology (GO) annotations of the *H. brasiliensis* RRIM 600 genome [28,31] were converted to the GO slim [31]. GO categories of the DEGs that were significantly enriched were identified using a hypergeometric test (*p* < 0.05). Only the biological process was focused on in this research.

Gene IDs in the RRIM 600 are registered at RIKEN rubber database [32].

### 2.3. Search for TF Genes Highly Expressed in Latex

Ten TF genes highly expressed in latex (Figure S1) were selected using previous tissue-specific RNA-seq analysis in four tissues (latex, petiole, bark, and leaf) of *H. brasiliensis* RRIM 600 [11], where significant differences were defined by Cuffdiff (*p* < 0.05) and a fold difference > 5. 

### 2.4. Phylogenetic Tree and Domain Search

The homology searches of the amino acid sequences of the bHLH TFs from *H. brasiliensis* and *Arabidopsis thaliana* were performed using Clustal X version 2.1 software. The phylogenetic tree was created using MEGA7 [33]. The domains were searched using the NCBI Conserved Domain Database.

### 2.5. Construction of Plasmids and In Vitro Protein Synthesis for gDB-seq

Coding sequences of HA epitope-tagged Hb_MYC2-1, Hb_MYC2-2, Hb_bHLH1, and Hb_bHLH2 were amplified by PCR from the cDNA pool using the gene-specific primers listed in Table S1 and inserted into the Xho I-Not I restriction enzyme sites of the pEU-His vector creating pEU_His-Hb_bHLH1-HA, pEU_His-Hb_bHLH2-HA, pEU_His-Hb_MYC2-1-HA, and pEU_His-Hb_MYC2-2-HA. The plasmids, except pEU_His-Hb_bHLH1-HA, were used as templates for in vitro transcription reactions. The template for Hb_bHLH1 transcription was amplified from the ligated pEU_His-Hb_bHLH1-HA plasmid using the SP6p and bHLH1_R2 primers listed in Table S1. 

Recombinant protein was synthesized in vitro through transcription and translation in wheat germ extract using WEPRO 7240H Expression Kits (CellFree Sciences, Yokohama, Japan) according to the middle reaction scale (1.2 mL) protocol of the manufacturer’s instructions. As a negative control, empty pEU-His vector was used for protein synthesis.

### 2.6. SDS-PAGE

SDS-PAGE has been described previously [26], except that non-purified crude recombinant proteins were subjected to electrophoresis in this research.

### 2.7. gDB-Seq Analysis

Genomic DNA (gDNA) was extracted from the cultured cells using DNeasy Plant Maxi Kits (QIAGEN, Hilden, German), according to the manufacturer’s instructions, and fragmented to approximately 150 bp in length using Covaris S2 (Covaris Ltd, Brighton, UK).

Five micrograms of anti-HA antibody (Fujifilm Wako, Osaka, Japan), 60 μL of Dynabeads Protein G (Invitrogen), 250 μL of recombinant proteins, and 250 μL of solution A (10 mM Tris-HCl (pH 7.5), 50 mM KCl, 0.2 mM MgCl_2_, 1 mM DTT, 2.5% glycerol) were mixed in a microtube, and the mixture was incubated rotating at 4 °C for 2 h. Thereafter, 150 μL of the recombinant protein, 2.5 μg of the anti-HA antibody, and 250 μL of solution A were added to the magnetically purified beads again, and the mixture was incubated rotating at 4 °C for 2 h. The protein-bound beads were washed three times with solution A, resuspended with 500 μL of solution A, mixed with 10 μg of the gDNA fragments and incubated rotating overnight at 4 °C. The beads were washed five times with solution A and resuspended in 200 μL of Proteinase K Buffer (10 mM Tris-HCl (pH 8.0), 10 mM EDTA, 0.5% SDS). Twenty micrograms of Proteinase K (Takara Bio, Kusatsu, Japan) were added to the tube followed by incubation at 37 °C for 2 h with occasional tapping. Twenty micrograms of Proteinase K were added again, and the mixture was incubated at 37 °C for 1 h. The bound gDNA fragments were extracted with phenol/chloroform treatment and ethanol precipitation.

The gDB-seq library was constructed using 5 ng of the bound gDNA fragments using a KAPA Hyper Prep Kit (Roche, Basel, Switzerland), almost following the manufacturer’s protocol. The one exception was that, after the post-ligation clean up, gDNA fragments of 200–500 bp were isolated using agarose gel electrophoresis and then purified using a MinElute Gel Extraction Kit (QIAGEN). Twenty-two PCR cycles were used for library amplification. 

The libraries were pair-end sequenced (2 × 150 bp) using a MiSeq (Illumina, San Diego, USA). After quality control (*Q* > 20), the reads were mapped onto the genome of *H. brasiliensis* RRIM 600 using Bowtie 2 version 2.3.4.1 [34]. JBrowse version 1.14.1 was used for visualization of the mapping result [35]. TF binding sites were detected using MACS2 version 2.1.0 [36]. Significant difference was defined by a *p*-value < 0.01 and peak scores ≥ 4.0. CAGE data for transcription start sites has been described previously [28,32].

### 2.8. Western Blot Analysis

Immunoprecipitated recombinant proteins described above were detected using Western blot analysis. The procedures for SDS-PAGE, blotting, and detection have been described previously [26].

### 2.9. Data Deposition

The data generated by next-generation sequencers were deposited in the DDBJ/EMBL/GenBank BioProject under accession number PRJNA629427.

## 3. Results

### 3.1. Time-Course RNA-Seq Analysis in Cells Treated with MeJA

**Figure 1:**
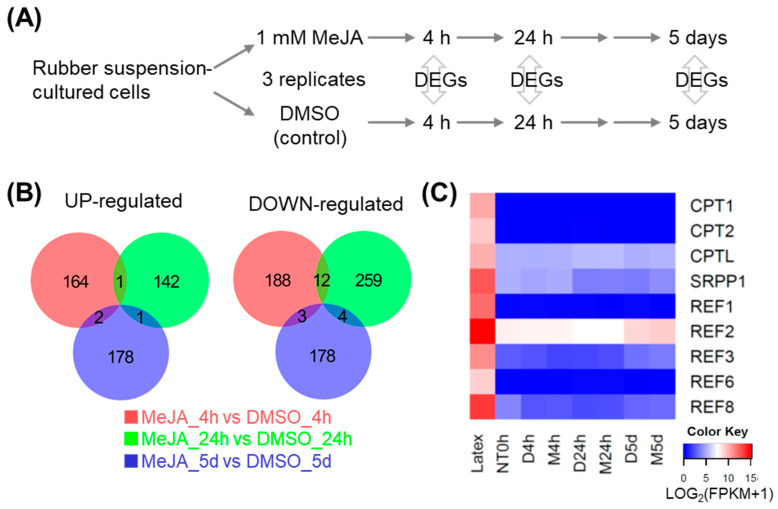
Time-course RNA-seq analysis of suspension-cultured cells upon MeJA treatment for 4 hours (h), 24 h, and 5 days (d). (**A**) Procedure of time-course RNA-seq analysis. Differentially expressed genes (DEGs) were identified by comparison between MeJA and DMSO (control) treatments at each time point (*p*-value < 0.05 and fold difference > 1.5 or <2/3). (**B**) Venn diagrams of the overlaps between up- or downregulated genes at 4 h, 24 h, and 5 days after MeJA treatment. (**C**) Heatmap of mRNA accumulation of nine rubber biosynthetic genes. The latex data were obtained from previous tissue-specific RNA-seq analysis [11]. NT = Non-treatment, D = DMSO, M = MeJA.

To investigate the effect of jasmonate on the transcriptome under uniform cell conditions, we carried out time-course RNA-seq analysis in suspension-cultured *H. brasiliensis* cells treated with MeJA. Samples were harvested at 0 hour (h), 4 h, 24 h, and 5 days (d) after addition of MeJA or dimethyl sulfoxide (DMSO). We selected these time points to see both early (4 h and 24 h) and late (5 d) responses to MeJA treatment. Differentially expressed genes (DEGs) were identified by comparing the MeJA and DMSO treatments at each time point (Figure 1A). At 4 h, 24 h, and 5 d after MeJA treatment, 203, 275, and 185 upregulated genes and 167, 144, and 181 downregulated genes were detected, respectively (Table 1 and Table S2). Overlaps of both upregulated and downregulated genes were fairly small between two of the three time points, and there is no overlap between the three (Figure 1B), indicating that the gene expression profile dramatically changed during the treatment period.

Our previous tissue-specific RNA-seq analysis detected the highest expression of major rubber biosynthesis genes, such as *CPT1*, *CPTL*, *SRPP1*, and *REF2*, in latex [11]. However, in this RNA-seq analysis, their expression was lower at all time points than in latex and none of them significantly changed during the treatment period (Figure 1C and Table S3). 

### 3.2. Characterization of the DEGs

**Figure 2:**
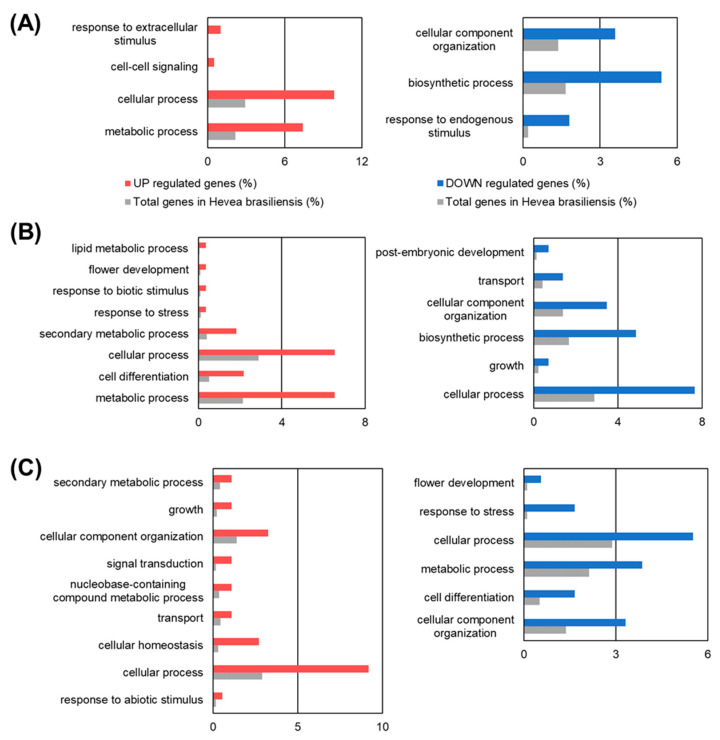
Gene Ontology (GO) enrichment analysis of DEGs. GO enrichment analysis was performed on genes whose expression was upregulated or downregulated upon MeJA treatment for 4 h (**A**), 24 h (**B**), and 5 d (**C**).

To know which biological processes were activated upon MeJA treatment, we performed GO analysis on the DEGs. The result showed that the term “cell differentiation” was enriched in upregulated genes at 24 h, but inversely, the term was enriched in downregulated genes at 5 d (Figure 2), indicating that MeJA induces cell differentiation into some kinds of cell type up to a few days after MeJA treatment.

Previous reports have suggested the possibility that some TFs participate in promoting laticifer differentiation and rubber biosynthesis by modulating transcription of their target genes [11,14,16]. In this research, upregulated genes at 4 h, 24 h, and 5 d after MeJA treatment include 15, 23, and 6 TF genes, respectively, while downregulated genes include 19, 9, and 12 TF genes, respectively (Table S4).

### 3.3. Hb_MYC2 and the Related bHLH TFs

**Figure 3:**
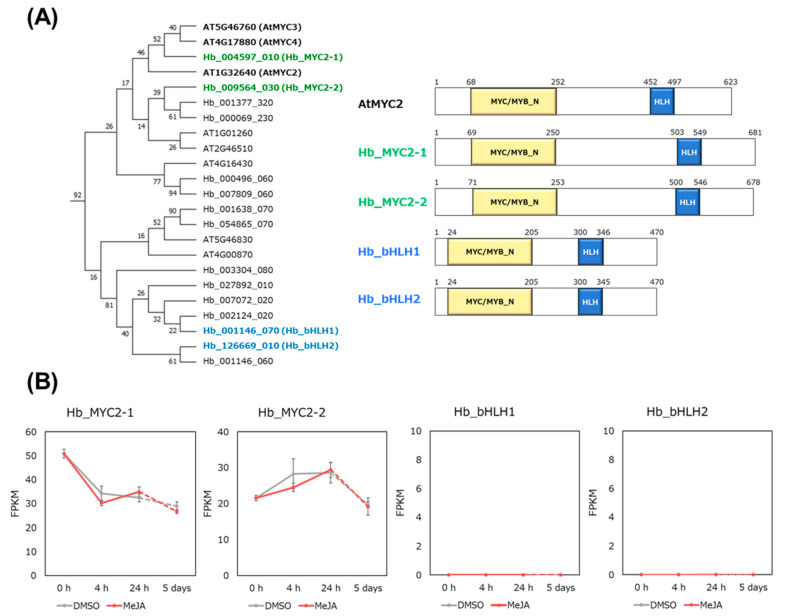
Characterization of four bHLH-type transcription factors (TFs), Hb_MYC2-1, Hb_MYC2-2, Hb_bHLH1, and Hb_bHLH2. (**A**) Phylogenetic tree of MYC2-related bHLH TFs from *Arabidopsis thaliana* and *Hevea brasiliensis* and the domain structures of four Hb_bHLH TFs. (**B**) Expression profiles of four bHLH TFs in time-course RNA-seq analysis upon MeJA or DMSO treatment.

Jasmonate signaling is positively regulated by MYC2 in *Arabidopsis* [18,19]. We recently searched for MYC2 homologues of *H. brasiliensis* in the RRIM 600 genome [28] and found two MYC2 homologues, Hb_MYC2-1 and Hb_MYC2-2, which have approximately 50% identity with AtMYC2 amino acids (Figure 3A). Hb_MYC2-1 is 87% identical with Hb_MYC2-2. 

Additionally, we selected two other MYC2-related bHLH-type TFs, Hb_bHLH1 and Hb_bHLH2, for the following analysis (Figure 3A), because previous tissue-specific RNA-seq analysis [11] showed that their expression was the highest in laticifer cells (latex) (Figure S1) compared with bark and stems. Hb_bHLH1 is 88% identical with Hb_BHLH2, although Hb_bHLH1 and Hb_bHLH2 showed approximately 30% identity with AtMYC2.

Time-course RNA-seq analysis showed that both *Hb_MYC2-1* and *Hb_MYC2-2* were expressed but neither *Hb_bHLH1* nor *Hb_bHLH2* were during the treatment period (Figure 3B). Taken together, it is easily assumed that the Hb_MYC2s may function in jasmonate signaling and the subsequent cell differentiation, and Hb_bHLH1 and Hb_bHLH2 may function in promoting rubber biosynthesis by activating transcription of rubber biosynthesis-related genes.

### 3.4. gDB-seq Analysis of Four bHLH TFs

**Figure 4:**
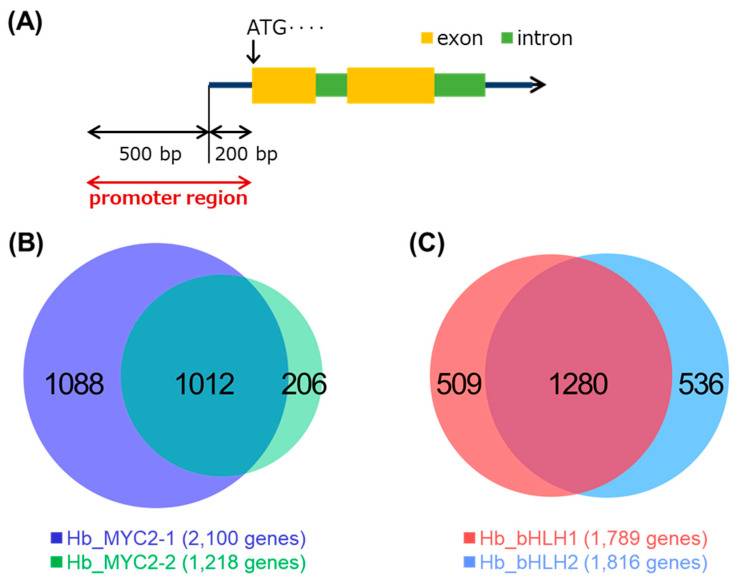
Identification of candidate genes targeted by four bHLH TFs using gDB-seq analysis. (**A**) Promoter regions are defined as being in the range 500 nt upstream to 200 nt downstream of the 5’ ends of genes. Genes, the promoters of which possess one or more binding sites, were selected as candidate genes targeted by the TFs. (**B**) and (**C**) Venn diagrams for the overlaps between candidate genes of Hb_MYC2-1 and Hb_MYC2-2 (B), and Hb_bHLH1 and Hb_bHLH2 (**C**).

To obtain information about the genes that are directly regulated by the four bHLH TFs, we performed gDB-seq analysis in which physical TF binding sites and target gene candidates were identified by sequencing genomic DNA fragments bound to in vitro-produced recombinant TFs (Figure S2). Here, a promoter was defined as being in the range 500 nt upstream to 200 nt downstream of the 5’ end of a gene (Figure 4A). Genes, the promoters of which possess one or more binding sites, were selected as candidates targeted by the TFs. Numbers of the detected binding sites and target candidate genes are shown in Table 2 and the candidate genes are listed in Table S5.

Candidate genes of MYC2-1 include 83% of those of MYC2-2 (Figure 4B) and overlap of the genes between Hb_bHLH1 and Hb_bHLH2 is approximately 70% (Figure 4C), indicating similar binding properties between MYC2-1 and MYC2-2, and between Hb_bHLH1 and Hb_bHLH2. 

### 3.5. DEGs Upon MeJA Include Candidate Genes Targeted by Hb_MYC2s 

Upregulated genes at 4 h, 6 h, and 5 d after MeJA treatment included 12, 20, and 9 candidate genes of MYC2-1, MYC2-2, or both, respectively, while downregulated genes included 5, 5, and 5 candidate genes, respectively. They include several kinds of TF genes such as ERF, WRKY, and the MYB family of TFs (Table 1 and Table S4). 

### 3.6. Candidate Genes of the Four TFs Include Rubber Biosynthesis-Related Ones

**Figure 5:**
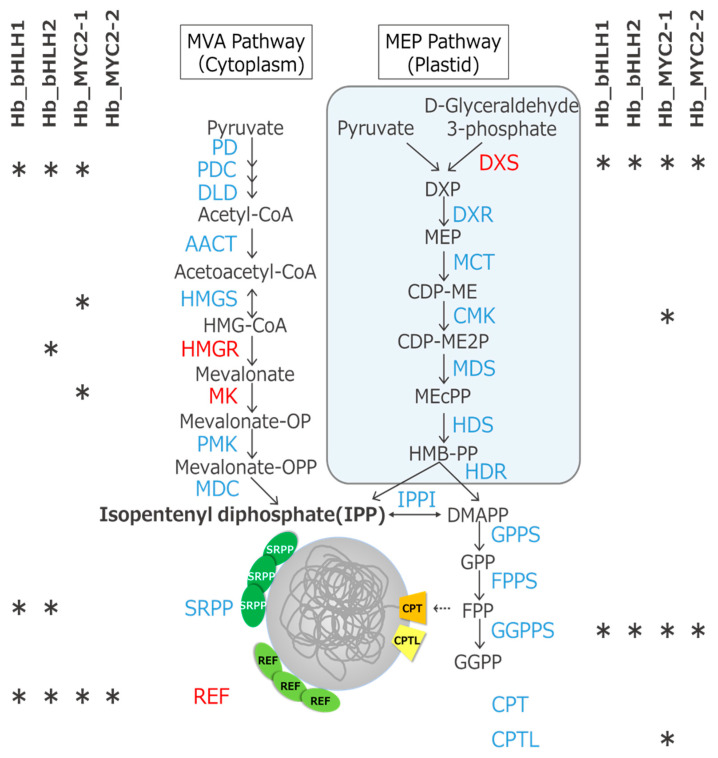
Candidate genes targeted by four bHLH-type TFs in the MVA, MEP, and rubber biosynthetic pathways. Asterisks indicate candidate genes. The family names (DXS, HMGR, MK, and REF) that include upregulated genes upon MeJA treatment are presented by red color. *DXS8* (Hb_012796_050), *HMGR5* (Hb_000116_530), and *MK3* (Hb_002042_080) and *REF7* (Hb_001741_110) were upregulated at 4 h, 24 h, and 5 d after MeJA treatment, respectively. Downregulated genes upon MeJA treatment were not found in these gene families.

Rubber biosynthesis is mediated by two steps: IPP production in the MVA and MEP pathways, and polymerization of IPPs into rubber on rubber particles [3]. We found that candidate genes targeted by the four TFs include a variety of MVA and MEP pathway genes and rubber biosynthetic genes (Figure 5 and Table S3). 

**Figure 6:**
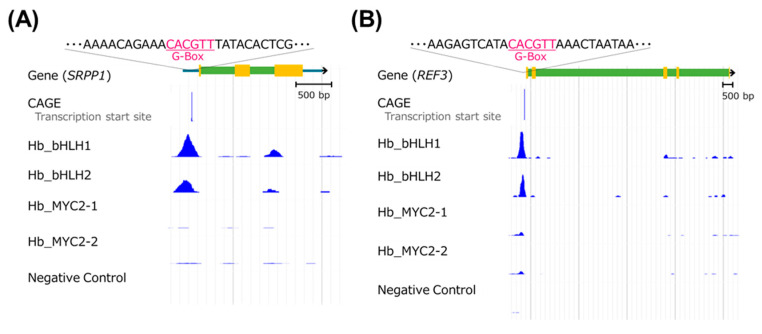
Genome browser view of *SRPP1* and *REF3* loci. Hb_bHLH1 and Hb_bHLH2 physically bind upstream of the transcription start sites (CAGE) of *SRPP1* (**A**) and *REF3* (**B**). Both binding regions contain a G-box sequence, which is the binding motif of bHLH TFs.

In particular, Hb_bHLH1 and Hb_bHLH2 physically bound to just upstream of the transcription start sites [28,32] of the *SRPP1* and *REF3* rubber biosynthetic genes (Figure 6). G-box sequences containing an ACGT core were seen in both binding peaks, indicating that these are the binding sites of the bHLH TFs [19]. 

## 4. Discussion

Our previous report showed that some rubber biosynthetic genes such as *SRPP1* and *REF3* highly express in latex (laticifer cells) [11]. In addition, *Hb_bHLH1* and *Hb_bHLH2* also highly express in latex (Figure S1). In this research using gDB-seq analysis, an in vitro DNA-protein binding assay, we present evidence that both TFs physically bind to the promoter regions just upstream of the transcription start sites of the *SRPP1* and *REF3* genes (Figure 6). This result suggests the strong possibility that Hb_bHLH1 and Hb_bHLH2 regulate natural rubber biosynthesis by activating transcription of these genes. 

Protein alignment analysis shows that Hb_bHLH1 and Hb_bHLH2 have great similarity to the Hb_MYC2s (Figure 3A). However, their roles in *H. brasiliensis* are distinct, because expression patterns of *Hb_bHLH1* and *Hb_bHLH2* are different from those of the *Hb_MYC2*s (Figure 3B and Figure S1). It is suggested that Hb_bHLH1 and Hb_bHLH2 act in promoting rubber biosynthesis, and the Hb_MYC2s in jasmonate signaling.

It is well known that jasmonate treatment to bark promotes secondary laticifer differentiation and later natural rubber biosynthesis [12]. Usage of a uniform cell line is important to examine the precise effect of jasmonate treatment on the gene expression profile. Here, time-course RNA-seq analysis using suspension-cultured cells upon MeJA treatment was performed to understand jasmonate signaling. GO analysis showed that the term “cell differentiation” was enriched in the upregulated genes at 24 h after MeJA treatment (Figure 2), indicating that MeJA could induce gene expression changes that were associated with the differentiation process. However, during the treatment period (5 days after MeJA treatment), significant changes in the expression of major rubber biosynthetic genes were not seen (Figure 1C), whereas the expression of these genes was highest in latex (laticifer cells) compared to several other tissues [11]. A previous report indicated that two months were needed following jasmonate treatment for differentiation from bark to laticifer cells to occur [16]. Taken together, MeJA may induce differentiation of the suspension cells, but they could not become laticifer cells until at least 5 d after MeJA treatment. In fact, we did not detect latex in the cells (our unpublished observation). Regrettably, it is difficult to culture the cells in the MeJA-containing medium for more than 5 d, because they gradually start to die after 5 d incubation.

AtMYC2 is a central regulator of jasmonate signaling in *Arabidopsis* [18,19]. It has been speculated that secondary laticifer differentiation is regulated by MYC2-mediated signaling in *H. brasiliensis* [23,37,38]. Our RNA-seq and gDB-seq analyses show that upregulated genes at 4 h, 24 h, and 5 d after MeJA treatment include 15, 23, and 6 TF genes, respectively, and the physical binding sites of two AtMYC2 orthologues, Hb_MYC2-1 and Hb_MYC2-2, were seen in the promoter regions of several of them (Table 1 and Table S4). A previous report showed that treating rubber trees with jasmonate induced transcription of TF genes encoding MYB, AP2, and the bHLH family [16], and high expression of TF genes encoding C2C2-Dof, TAZ, and the AP2 family together with the bHLH family was observed in latex (laticifer cells) compared with other tissues [11]. Multiple TFs and their interactors orchestrate to function in biological processes, such as jasmonate signaling, to form a TF network [24,39,40,41]. Therefore, it is considered that MYC2-triggered activation of a TF network also plays an important role in the jasmonate response and subsequent expected cell differentiation in *H. brasiliensis*. 

There have been a few reports that identified candidate genes targeted by TFs in *H. brasiliensis*. Yang et al. showed that HbEIN3 could bind to the promoter of the *CPT1* rubber biosynthetic gene [42]. In addition, Zhai et al. showed that Hb_MYC2 could bind to the promoter of *HbPIP2;1* [38]. They used indirect detection methods, such as yeast survival tests and/or in trans reporter assays, but not any direct methods. In this report, we have identified candidate genes throughout the genome that are targeted by Hb_MYC2s using gDB-seq, which enables us to know the direct TF binding sites in vitro. Thus, in vitro methodologies like gDB-seq are very useful for non-model woody crops like *H. brasiliensis*, in which it may be hard to perform chromatin-immunoprecipitation sequencing (ChIP-seq) analysis for detecting in vivo TF binding.

bHLH-type TFs bind to DNA by forming homo- or heterodimers [43,44], but gDB-seq identifies only homodimer binding sites [25,26]. In order to obtain information of all binding sites of both homo- and heterodimers and reveal the complete TF network for rubber biosynthesis, further identification of candidate genes targeted by each TF using both gDB-seq and ChIP-seq analyses will be required in the future. 

## 5. Conclusions

Natural rubber is an irreplaceable material obtained industrially only from *H. brasiliensis*. Understanding its biosynthesis pathway is required in order to increase the yield. Here, we present novel insights into associations between the changes in the gene expression profile upon MeJA treatment and the bHLH-type TFs, MYC2s, and between the expression of two rubber biosynthetic genes, *SRPP1* and *REF3*, and other bHLH-type TFs, bHLH1 and bHLH2. We hope that, in the future, our findings will help to increase the yield of natural rubber through genetic control.

## Figures and Tables

**Table 1:** Summary of time-course RNA-seq analysis.

Treatment Time		4 h	24 h	5 Days
UP-regulated genes		203	275	185
TF	UP-regulated	15	23	6
	UP-regulated	NAC	ERF, WRKY, MYB, Tify, MYB-related	―
	&			
	Hb_MYC2 binding			
Rubber biosynthesis-related	UP-regulated	1	1	2
	UP-regulated	―	―	MK3, REF7
	&			
	Hb_MYC2 binding			
DOWN-regulated genes		167	144	181
TF	DOWN-regulated	19	9	12
	DOWN-regulated	C2C2-GATA	M-type	―
	&			
	Hb_MYC2 binding			
Rubber biosynthesis-related	DOWN-regulated	0	0	0
	DOWN-regulated	―	―	―
	&			
	Hb_MYC2 binding			

**Table 2:** Summary of gDB-seq analysis.

Sample	Number of Peaks	Number of Peaks in Promoter Regions	Number of TF-Targeted Candidate Genes
Hb_MYC2-1	39,429	2105	2100
Hb_MYC2-2	24,291	1219	1218
Hb_bHLH1	20,809	1797	1789
Hb_bHLH2	18,995	1823	1816

## Supplementary Materials

The following are available online at https://www.mdpi.com/2223-7747/9/6/674/s1, Figure S1: Heatmap of mRNA accumulation of 10 selected TF genes in tissue-specific RNA-seq analysis, Figure S2: Detection of recombinant TF proteins by SDS-PAGE and Western blot analysis; Table S1: Primers used in this work, Table S2: List of DEGs, Table S3: Rubber biosynthesis-related genes, Table S4: TFs included in DEGs, Table S5: List of candidate genes targeted by each TF.
