# Supplementary material for: Regulatory Potential of bHLH-Type Transcription Factors on the Road to Rubber Biosynthesis in Hevea brasiliensis
Source: Plants (Basel). 2020 May 26;9(6):674. doi: 10.3390/plants9060674 (PMC7355734; doi:10.3390/plants9060674)
Supplement: Supplementary file 1 [file plants-09-00674-s001.zip › Supplementary_data/Supplementary_Figures.pdf]

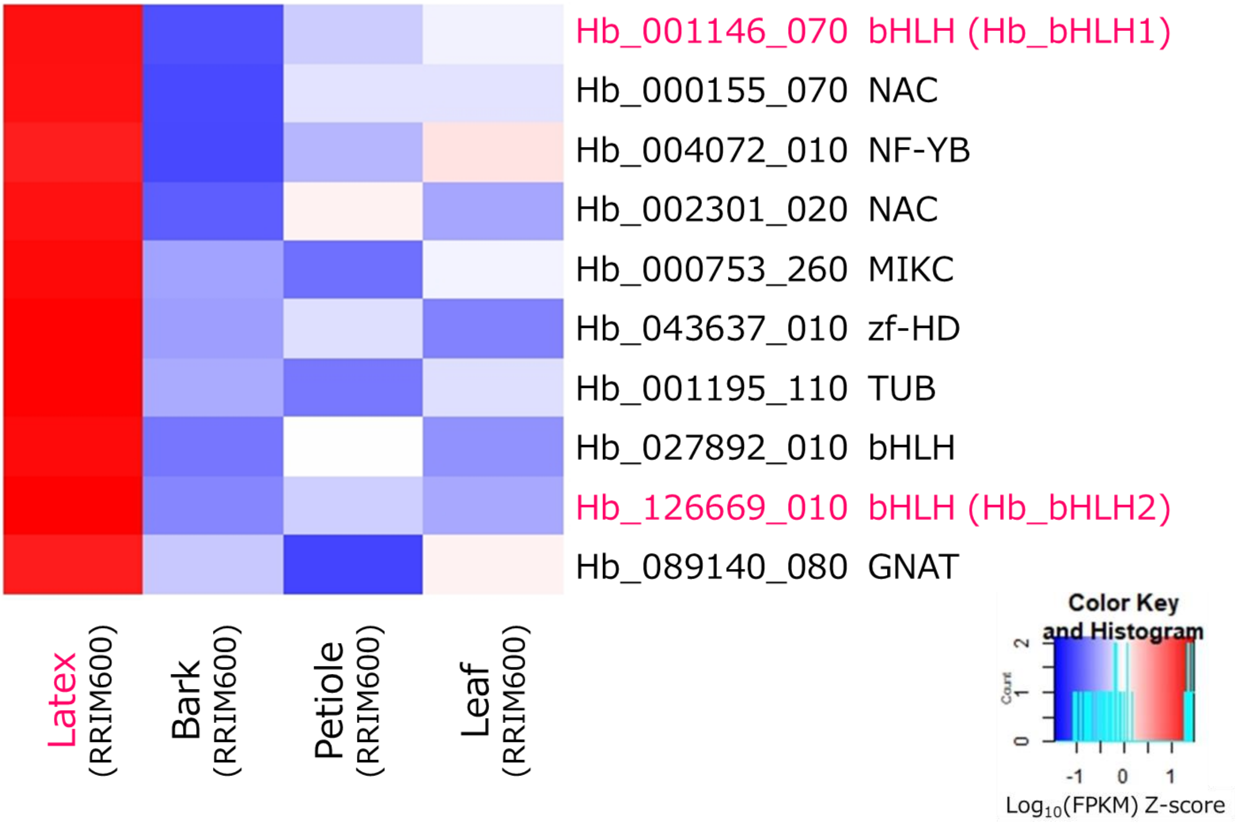

Figure S1. Heatmap of mRNA accumulation of 10 selected TF genes in tissue-specific RNA-seq analysis (Makita et al., 2017). They are highly expressed in latex. The TF family names are shown with the Gene IDs.

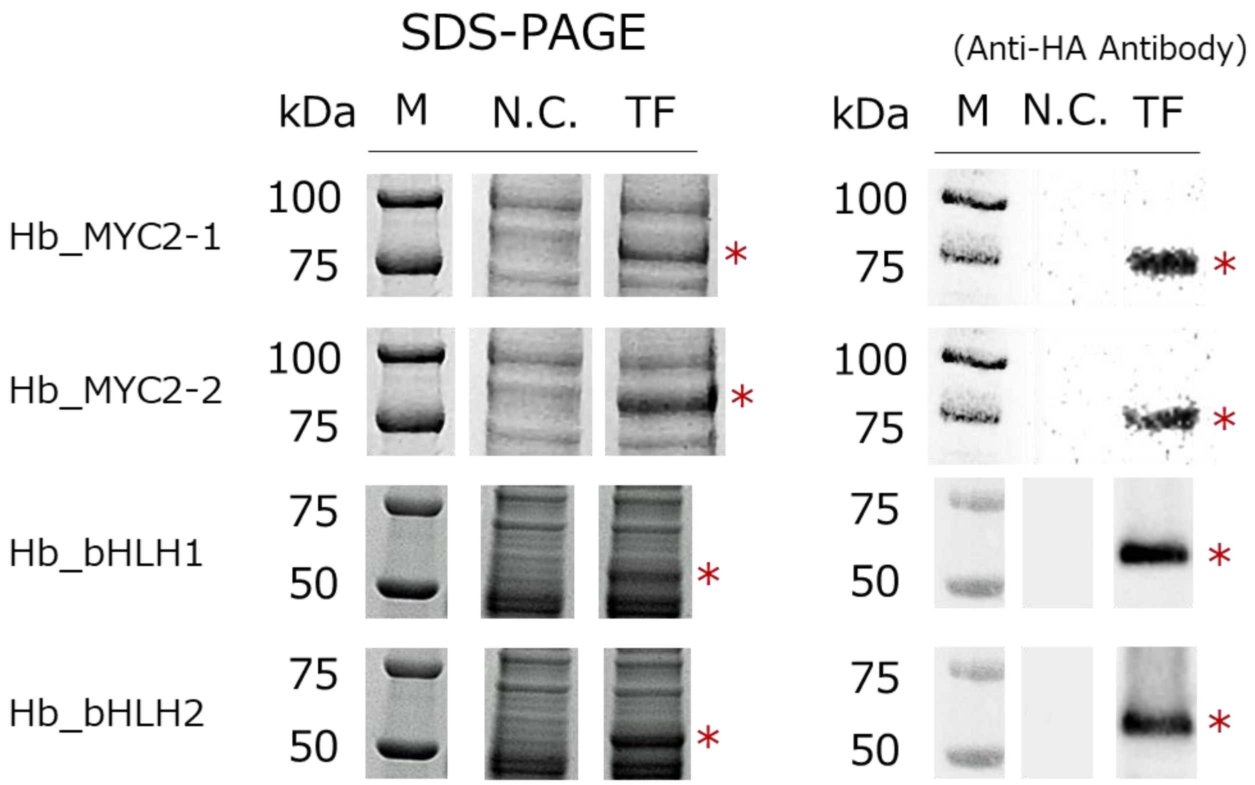

Figure S2. Detection of recombinant TF proteins by SDS-PAGE and western blot analysis. Recombinant proteins were synthesized in wheat germ extract. Asterisks indicate positions of recombinant proteins.
